# Supplementary material for: Hippocampal CA2 neurons disproportionately express AAV-delivered genetic cargo
Source: bioRxiv. 2024 Nov 28:2024.11.27.625768. Preprint. [Version 1] doi: 10.1101/2024.11.27.625768 (PMC11623684; doi:10.1101/2024.11.27.625768)
Supplement: Supplement 3 [file NIHPP2024.11.27.625768v1-supplement-3.pdf]

bioRxiv preprint doi: https://doi.org/10.1101/2024.11.27.625768; this version posted November 28, 2024. The copyright holder for this preprint (which was not certified by peer review) is the author/funder. This article is a US Government work. It is not subject to copyright under 17 USC 105 and is also made available for use under a CC0 license.

(subfield: F(3,30)=199.8, $p$<0.0001; genotype: F(1,10)=1.13, $p$=0.31; interaction: F(3,30)=0.84, $p$=0.48, RM two-way ANOVA). D. Immunostaining for FGFR1, which has been described as an AAV co-receptor, in tissue from C57BL/6J animals injected with PHP.B-hSyn-GFP shows colocalization of FGFR1 and GFP. Fluorescence intensity for FGFR1 was significantly greater in CA2 than all other subfields, and CA3 showed increased expression relative to CA1 and DG (F(3,33)=166.9, $p$<0.0001, RM one-way ANOVA; results of Tukey's multiple comparisons tests shown on graph). E. FGFR1 expression in CA2 is significantly decreased in Cre+ MR fl/fl aninmals compared to Cre- animals (subfield: F(3,33)=31.93, $p$<0.0001; genotype: F(1,11)=5.31, $p$=0.041; interaction: F(3,33)=26.44, $p$<0.0001; Bonferroni's multiple comparisons results shown on graph). F. FGFR1 expression is similar in all subfields between AAVR KOs and WT littermates (subfield: F(3,30)=167.7, $p$<0.0001; genotype: F(1,10)=0.048, $p$=0.83; interaction: F(3,30)=0.14, $p$=0.93, RM two-way ANOVA). Scale bar in A, D=200 $\mu$m. Scale bars in B, C, E, F = 100 $\mu$m. *p<0.05, ***p<0.001, ****p<0.0001.

Figure 8. Focal injection of AAV6-hSyn-GFP into CA2 permits GFP expression in EMX Cre+; MR fl/fl animals at levels not significantly different from Cre- animals. Mossy fibers are immunopositive for ZnT3, and CA1 pyramidal cells are immunopositive for WFS1. In Cre- animals, CA2 pyramidal cells reside between the mossy fiber stain and the WFS1 stain, but in Cre+ animals, ZnT3 and WFS overlap in CA2. Fluorescence intensity of GFP in CA2 delivered by AAV6 intrahippocampal injection was not significantly different between Cre- and Cre+ animals (t(12)=0.29, $p$=0.77, two-tailed unpaired t-test).

Supplemental Figure 1. Intrahippocampally injected AAVs showed differential preference for CA2 expression according to serotype. GFP fluorescence intensity was measured in each hippocampal subfield at each of the injection site (-2.3 mm AP) and a site in dorsal hippocampus (-1.8 mm AP). Image acquisition settings were held constant within each serotype but differed across serotypes. Fluorescence intensity values were compared using two-way ANOVAs with Sidak's multiple comparisons tests. Main effects and interactions are as follows and results of Sidak's multiple comparison's tests are shown on each graph. AAV1: main effect of AP level: F(1,3)=5.40, $p$=0.10, main effect of subfield: F(3,9)=5.38, $p$=0.021, interaction: F(3,9)=1.10, $p$=0.40. AAV2: AP level: F(1,3)=0.99, $p$=0.39, subfield: F(3,9)=14.34, $p$=0.0009, interaction: F(3,9)=42.28, $p$<0.0001. AAV5: AP level: F(1,3)=0.0009, $p$=0.98, subfield: F(3,9)=9.39, $p$=0.0039, interaction: F(3,9)=11.44, $p$=0.0020. AAV6: AP level: F(1,3)=33.18, $p$=0.010, subfield: F(3,9)=22.59, $p$=0.0002, interaction: F(3,9)=29.27, $p$<0.0001. AAV8: AP level: F(1,3)=0.51, $p$=0.52, subfield: F(3,9)=5.38, $p$=0.021, interaction: F(3,9)=11.78, $p$=0.0018. AAV9: AP level: F(1,5)=0.61, $p$=0.47, subfield: F(3,15)=13.76, $p$=0.0001; interaction: F(3,15)=18.26, $p$<0.0001. AAVDJ: AP level: F(1,3)=6.92, $p$=0.078, subfield: F(3,9)=24.03, $p$=0.0001, interaction: F(3,9)=22.89, $p$=0.0002.

Supplemental Figure 2. CAP-B10-hSyn-jGCaMP8s shows prominent tropism for CA2 neurons. Statistics are not included due to the small number of animals (N=2 mice).

Supplemental Figure 3. Aggrecan expression is significantly decreased in Amigo2 Cre+ *Acan* fl/fl animals. Expression on brevican, neurocan and versican are not affected by

bioRxiv preprint doi: https://doi.org/10.1101/2024.11.27.625768; this version posted November 28, 2024. The copyright holder for this preprint (which was not certified by peer review) is the author/funder. This article is a US Government work. It is not subject to copyright under 17 USC 105 and is also made available for use under a CC0 license.

*Acan* deletion in CA2 (Aggrecan: subfield: $F(2,26)=491.7$, $p<0.0001$; genotype: $F(1,13)=283.1$, $p<0.0001$; interaction: $F(2,26)=468.2$, $p<0.0001$; Brevican: subfield: $F(2,10)=82.21$, $p<0.0001$; genotype: $F(1,5)=4.9\times10^{-5}$, $p=0.9948$; interaction: $F(2,10)=4.70$, $p=0.036$; Neurocan: subfield: $F(2,10)=46.04$, $p<0.0001$; genotype: $F(1,5)=1.15$, $p=0.33$; interaction: $F(2,10)=1.25$, $p=0.33$; Versican: subfield: $F(2,16)=36.91$, $p<0.0001$; genotype: $F(1,8)=0.58$, $p=0.47$; interaction: $F(2,16)=0.64$, $p=0.54$. Scale bar = 250 μm.

Supplemental Figure 4. A. MAL I, which recognizes terminal galactose glycans shows light stain on the cell membrane of CA2 pyramidal cells, encircling PCP4-positive cells Grayscale image below shows more clearly than color used for colocalization. MAL I staining was significantly higher in CA2 than either CA1 or CA3 ($F(2,6)=171.8$, $p<0.0001$; one-way ANOVA). B-C. MAL I staining appeared decreased in Cre+ MR fl/fl animals (B) but appeared similar to that in C57BL/6J animals (C). D. The PHP receptor, LY6A, on vasculature does not appear to be enriched near CA2. Scale bars = 100 μm in A, 250 μm in B.

Supplemental Video 1. Expression of GFP in an Amigo2 CreERT2; ROSA tdTomato mouse following retro-orbital injection of PHP.B-hSyn-GFP. CA2 pyramidal cells and their projections are shown in red.

Supplemental Video 2. Expression of GFP (pseudocolored as “fire”) in an Amigo2 Cre+; *Acan* fl/fl animal.

bioRxiv preprint doi: https://doi.org/10.1101/2024.11.27.625768; this version posted November 28, 2024. The copyright holder for this preprint (which was not certified by peer review) is the author/funder. This article is a US Government work. It is not subject to copyright under 17 USC 105 and is also made available for use under a CC0 license.

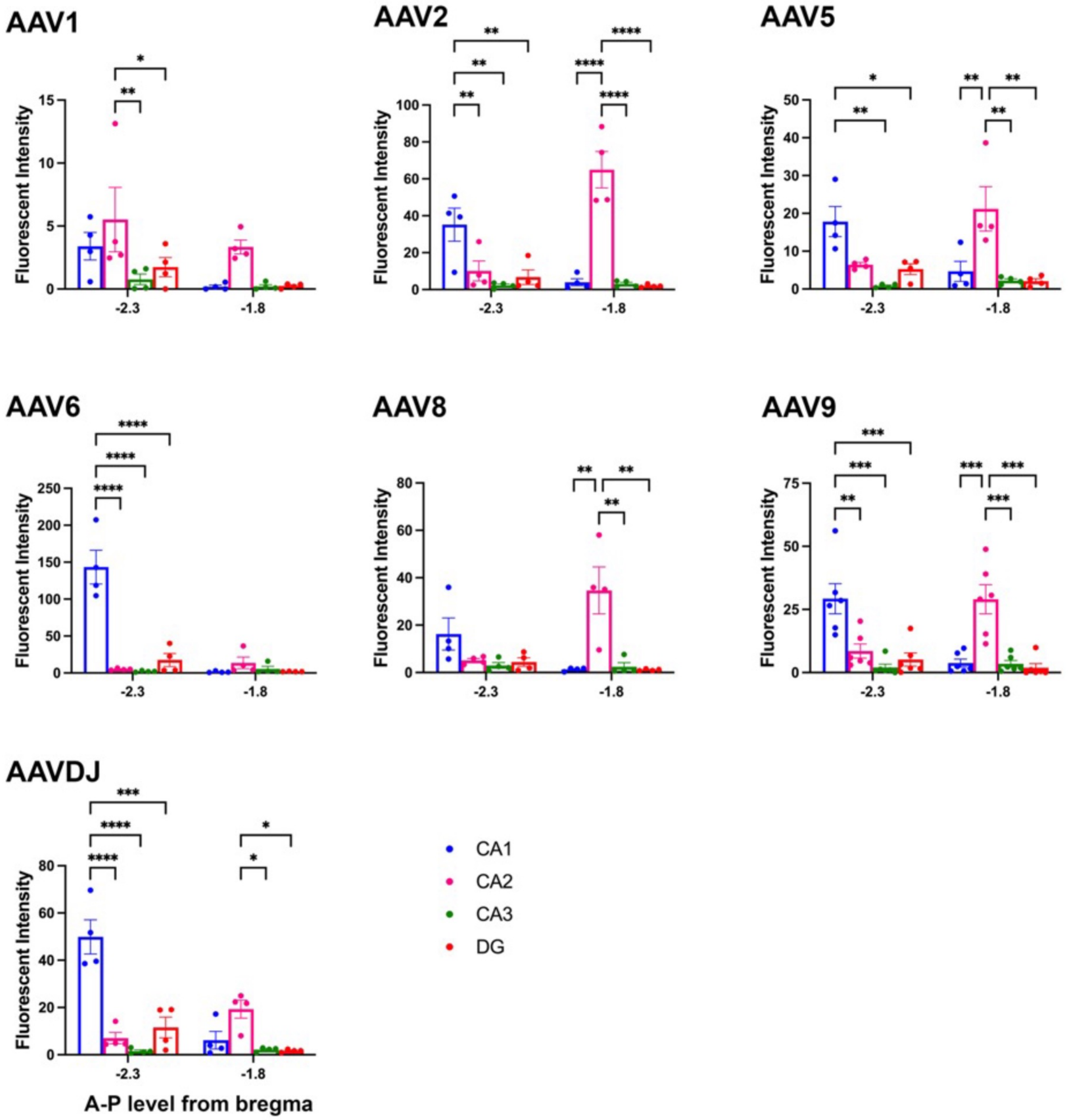


Supplemental Figure 1

bioRxiv preprint doi: https://doi.org/10.1101/2024.11.27.625768; this version posted November 28, 2024. The copyright holder for this preprint (which was not certified by peer review) is the author/funder. This article is a US Government work. It is not subject to copyright under 17 USC 105 and is also made available for use under a CC0 license.

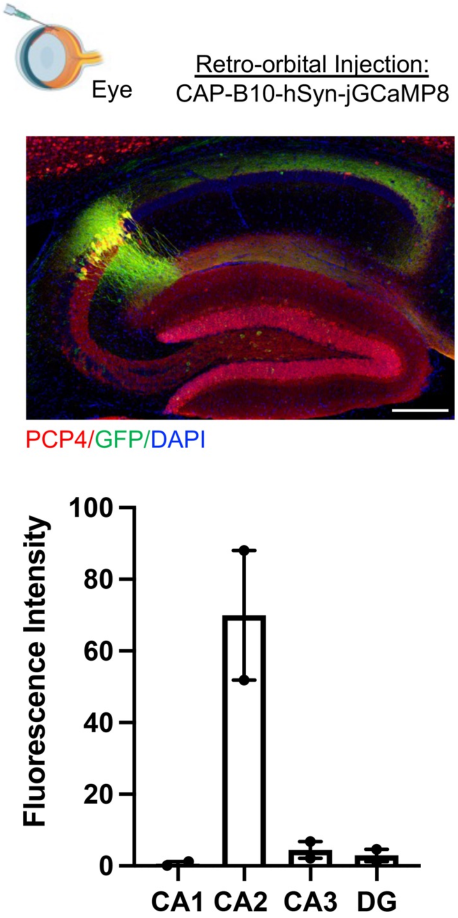


Supplemental Figure 2

bioRxiv preprint doi: https://doi.org/10.1101/2024.11.27.625768; this version posted November 28, 2024. The copyright holder for this preprint (which was not certified by peer review) is the author/funder. This article is a US Government work. It is not subject to copyright under 17 USC 105 and is also made available for use under a CC0 license.

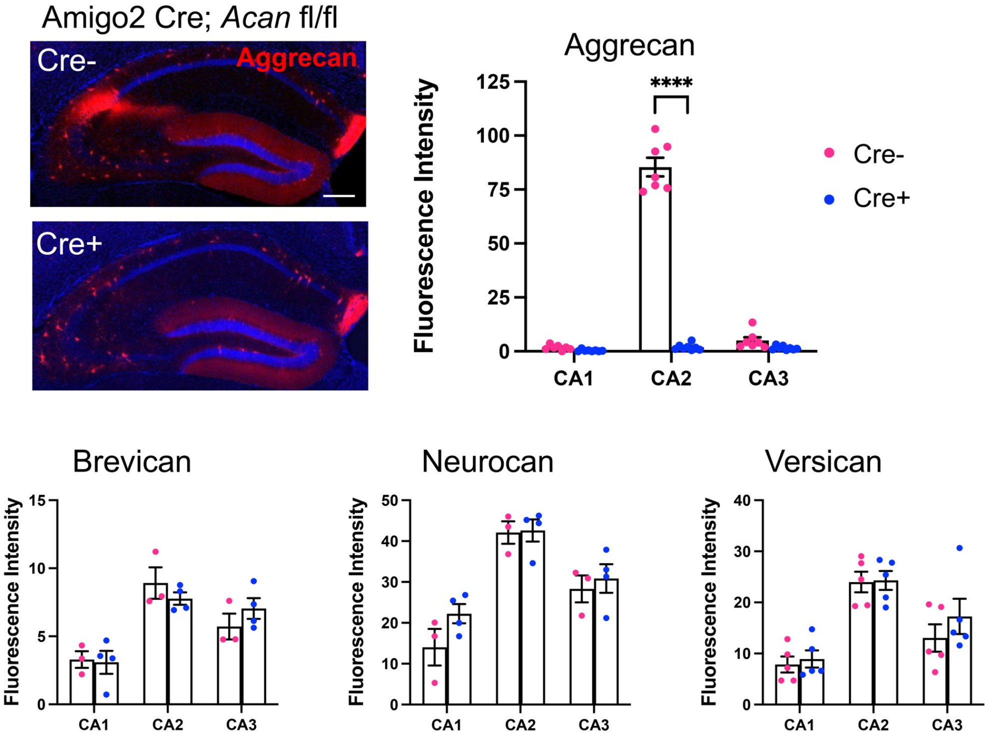


Supplemental Figure 3

bioRxiv preprint doi: https://doi.org/10.1101/2024.11.27.625768; this version posted November 28, 2024. The copyright holder for this preprint (which was not certified by peer review) is the author/funder. This article is a US Government work. It is not subject to copyright under 17 USC 105 and is also made available for use under a CC0 license.

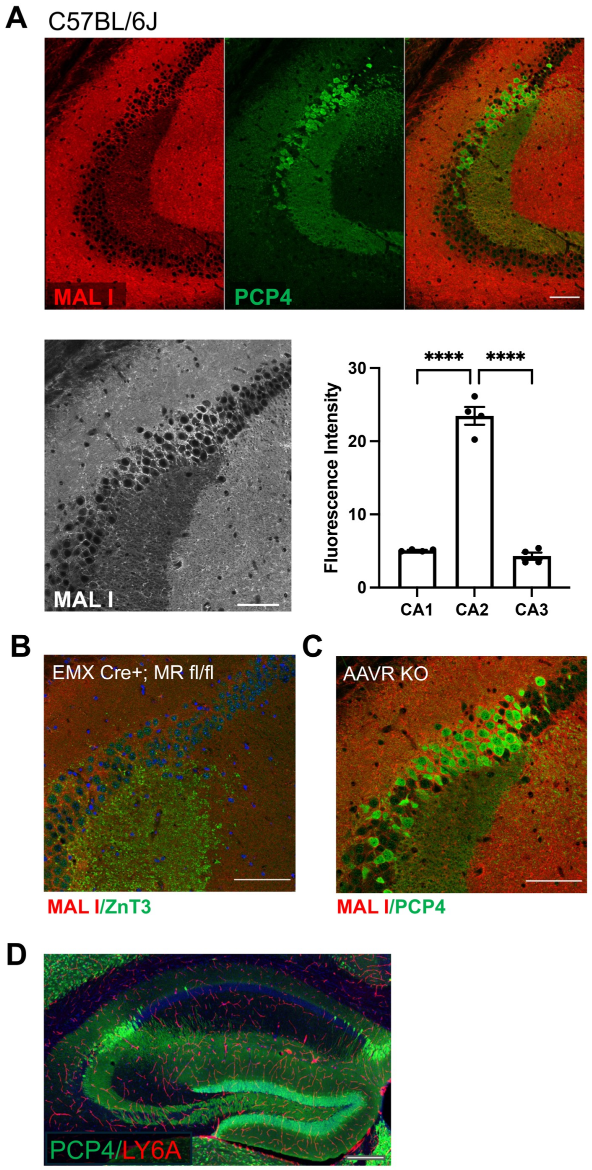


Supplemental Figure 4
